# Uma Forma Complicada de Infarto de “Um Segmento” Miocárdico: O Papel da Imagem Cardiovascular

**DOI:** 10.36660/abc.20190323

**Published:** 2020-09-11

**Authors:** Ana Rita Pereira, Ana Rita Almeida, Inês Cruz, Luis Rocha Lopes, Maria José Loureiro, Hélder Pereira

**Affiliations:** 1 Serviço de Cardiologia Hospital Garcia de Orta EPE Almada Portugal Serviço de Cardiologia, Hospital Garcia de Orta EPE, Almada - Portugal; 2 Barts Heart Centre Londres Inglaterra Barts Heart Centre, Barts Health NHS Trust, Londres - Inglaterra; 3 Institute of Cardiovascular Science University College London Londres Inglaterra Institute of Cardiovascular Science, University College London,Londres - Inglaterra; 4 Centro Cardiovascular Centro Académico de Medicina de Lisboa Universidade de Lisboa Lisboa Portugal Centro Cardiovascular, Centro Académico de Medicina de Lisboa, Universidade de Lisboa, Lisboa - Portugal

**Keywords:** Ruptura Cardíaca, Infarto do Miocárdio, Falso Aneurisma, Diagnóstico por Imagem, Ecocardiografia/métodos

## Introdução

A incidência de complicações mecânicas (CM) após o infarto do miocárdio (IM) foi reduzida para menos de 1% com o uso rotineiro de terapias de reperfusão primária.^1^ As CM são classificadas como precoces, incluindo as formas agudas e subagudas, e tardias ou crónicas.^2^ As primeiras apresentam-se principalmente como choque cardiogênico^2^ e as últimas podem variar de assintomáticas a apresentação com morte súbita.^3^ Como todas essas condições podem ter consequências potencialmente letais, são necessários diagnóstico e tratamento oportunos.^1 - 3^

## Relato de Caso

Uma mulher de 57 anos de idade e fumante foi admitida no Serviço de Emergência com dor no peito anterior opressiva, náusea e vômito. Quatro dias antes, a paciente reportou sintomas semelhantes com horas de evolução, mas alívio espontâneo. Durante a admissão, ela estava consciente e apresentava dor no peito. O exame médico revelou hipotensão, taquicardia, polipneia e sinais de diminuição da perfusão periférica.

**Figura 1:**
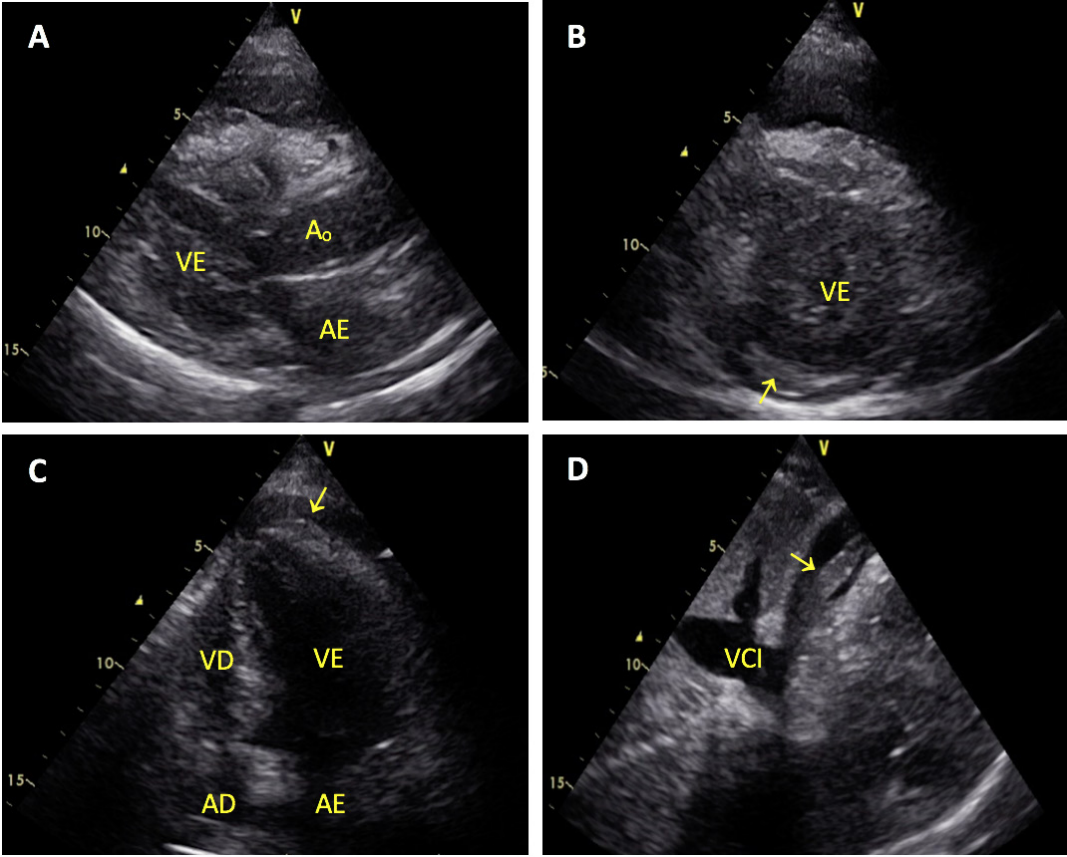
– Ecocardiograma transtorácico. (A) Janela paraesternal longo eixo e (B) Janela paraesternal curto eixo mostrando um ventrículo esquerdo ligeiramente hipertrofiado e não dilatado; (C) Janela apical 4 câmaras mostrando colapso parcial das cavidades direitas e hipocinesia da parede lateral, mas função sistólica do ventrículo esquerdo preservada; (D) Janela subcostal mostrando veia cava inferior dilatada, sem variação respiratória. A seta amarela indica derrame pericárdico moderado. VE: ventrículo esquerdo; AE: átrio esquerdo; Ao; aorta ascendente; VD: ventrículo direito; AD: átrio direito; VCI: veia cava inferior.

Um eletrocardiograma de 12 derivações mostrou taquicardia sinusal com elevação do segmento ST de 4 mm nas derivações DI e aVL e depressão do segmento ST de 4 mm nas derivações inferiores. Exames complementares revelaram acidose láctica, parâmetros inflamatórios sistêmicos elevados e aumento dos marcadores de necrose miocárdica. O ecocardiograma transtorácico (ETT) demonstrou ventrículo esquerdo hipertrofiado e não dilatado, com hipocinésia da parede lateral, mas com função sistólica preservada; derrame pericárdico moderado com colapso diastólico parcial das cavidades direitas; e veia cava inferior dilatada sem variação respiratória ( Figura 1 ; Vídeo 1). Não houve achados valvulares significativos e a raiz e o arco aórtico estavam normais.

**Vídeo 1:** – Ressonância magnética cardíaca de 4 câmaras (à esquerda) e 3 câmaras (à direita). URL: http://abccardiol.org/supplementary-material/2020/11503/2019-0853_video01.mp4

Por suspeita de infarto agudo do miocárdio com supradesnivelamento do segmento ST (IAMCSST) complicado por ruptura da parede livre (RPL) ventricular esquerda (VE), não foi administrada medicação antitrombótica e a paciente foi submetida a angiografia coronária invasiva (ACI) e ventriculografia (Vídeo 2). Observou-se estenose de 90% do ramo póstero-lateral, mas aparentemente não foram encontradas nenhuma lesão oclusiva, ruptura ventricular ou alterações da contractilidade segmentar do ventrículo esquerdo.

**Vídeo 2:** – Ventriculografia não mostrando ruptura ventricular ou alterações da contratilidade segmentar do ventrículo esquerdo. URL: http://abccardiol.org/supplementary-material/2020/11503/2019-0853_video02.mp4

**Figura 2:**
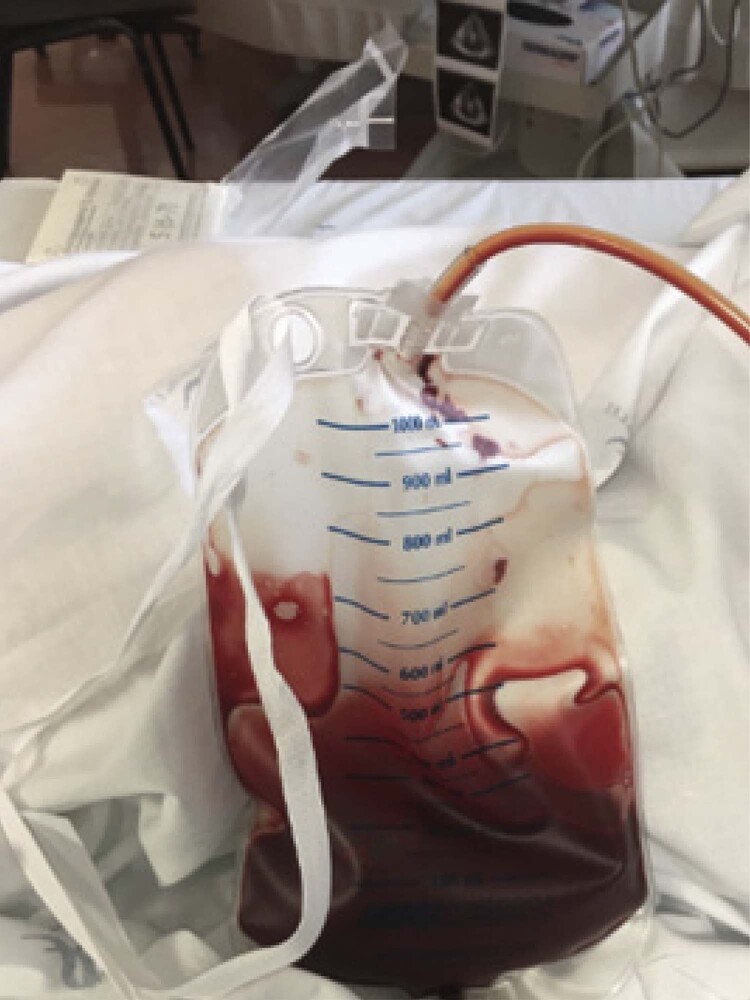
– Sistema de drenagem de pericardiocentese percutânea com cerca de 200 mL de líquido pericárdico hemático.

Após angiografia, seu quadro clínico piorou. Foi admitido tamponamento cardíaco e realizada pericardiocentese percutânea de emergência com drenagem de 200 mL de líquido hemático sem coagulação espontânea, resultando em melhora global ( Figura 2 ). A análise do fluído revelou tratar-se de um exsudado com adenosina desaminase normal. As análises microbiológicas foram negativas e o teste citológico não revelou células neoplásicas. Com o objetivo de determinar a etiologia do derrame, também foram realizadas sorologias virais, de autoimunidade e tomografia computadorizada toracoabdominal-pélvica com resultados normais.

**Figura 3:**
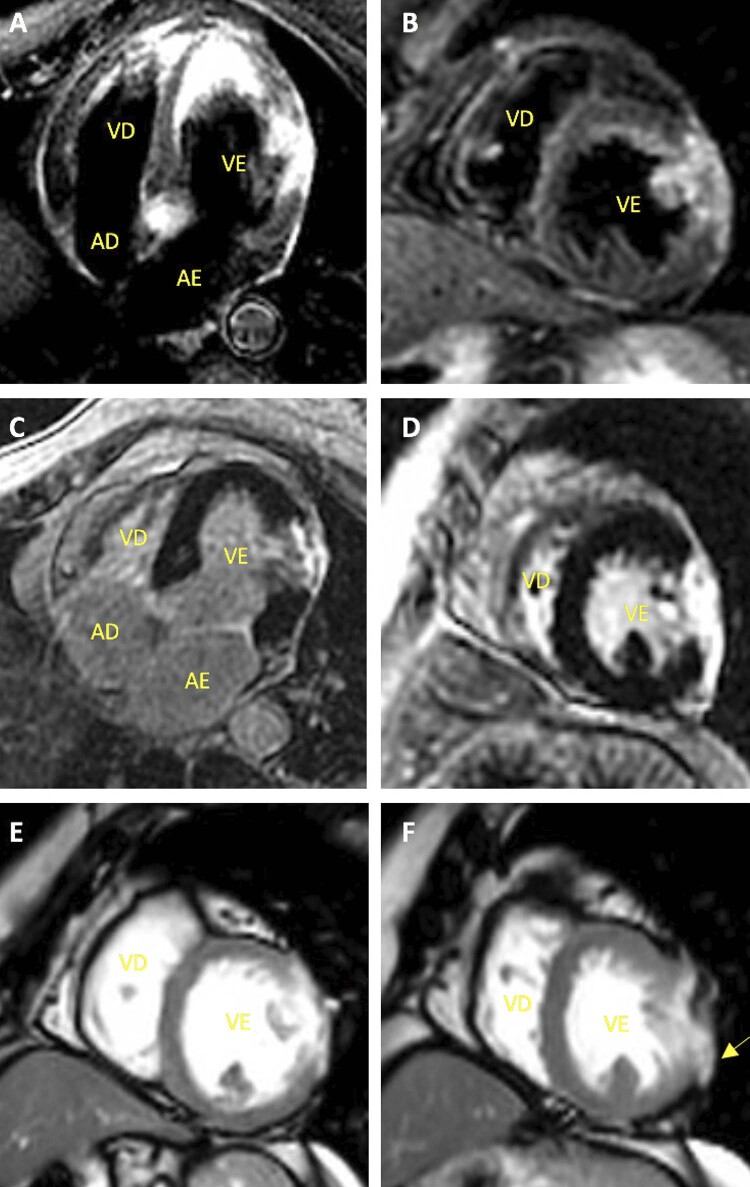
– Ressonância magnética cardíaca. (A) Imagem de quatro câmaras e (B) Imagem de eixo curto, sequências de recuperação de inversão de tau curta ponderadas em T2 (T2-weighted short-tau inversion recovery images, STIR), mostrando sinal hiperintenso transmural do segmento médio da parede lateral compatível com edema; (C) Imagem de quatro câmaras e (D) Imagem de eixo curto, sequências de realce tardio miocárdico com gadolínio, mostrando realce transmural do mesmo segmento, sugerindo necrose miocárdica; (E) fase telediastólica e (F) fase telesistólica, sequências de cine obtidas por precessão livre no estado estacionário (steady-state free precession, SSFP), mostrando discinesia do segmento médio da parede lateral e uma protuberância sacular sugerindo pseudoaneurisma (seta amarela).VE: ventrículo esquerdo; AE: átrio esquerdo; VD: ventrículo direito; AD: átrio direito.

Dada a ausência de um diagnóstico específico, foi realizada uma ressonância magnética cardíaca (RMC) oito dias após a internação. Revelou discinesia do segmento médio da parede lateral nas sequências de cine, sinal hiperintenso transmural nas imagens de recuperação de inversão de tau curta ponderada em T2 ( Figura 3A e 3B ) — compatíveis com edema — e realce tardio transmural ( Figura 3C e 3D ) — sugerindo necrose miocárdica — desse segmento. Esses achados foram compatíveis com IM subagudo do segmento médio da parede lateral, sem viabilidade aparente. Além disso, observou-se a ausência de tecido miocárdico entre os segmentos médios das paredes lateral e inferolateral, cercados por uma pequena protuberância sacular com pescoço estreito, sugerindo um pseudoaneurisma nesse local ( Figura 3E e 3F ; Vídeo 3).

**Vídeo 3:** – Ressonância magnética cardíaca, sequências de cine obtidas por precessão livre no estado estacionário (steady-state free precession, SSFP), vistas sequenciais em eixo curto, quatro câmaras e três câmaras, mostrando discinesia do segmento médio da parede lateral e protuberância sacular entre os segmentos médios da parede lateral e inferolateral, sugerindo um pseudoaneurisma.. URL: http://abccardiol.org/supplementary-material/2020/11503/2019-0853_video03.mp4

Assim, confirmou-se o diagnóstico inicialmente suspeito: IAMCSST subagudo complicado com RPL VE que evoluiu para tamponamento cardíaco e posteriormente para formação de pseudoaneurisma. Devido ao risco de complicações fatais, a paciente foi submetida a cirurgia cardíaca. Sem necessidade de circulação extracorpórea, foi realizada cirurgia de revascularização do miocárdio com enxerto de veia safena na artéria posterolateral e plicatura de pseudoaneurisma. Atualmente, a paciente encontra-se assintomática.

## Discussão

A RPL é uma CM incomum e precoce do IM, com incidência relatada inferior a 1%.^2^ Existem dois grupos clínicos: o “tipo *blow-out* ” (ruptura completa ou aguda) com defeito macroscópico e sangramento de alto volume, levando a tamponamento cardíaco; e o “tipo oozing” (ruptura incompleta ou subaguda) sem uma fonte óbvia de sangramento e acúmulo lento de sangue.^2^ O último tipo corresponde a até um terço dos casos e pode progredir para ruptura completa ou para formação de pseudoaneurisma.^1 - 3^ Em ambos tipos, a cirurgia imediata é vital, já que a RPL tem uma taxa de mortalidade entre 60 e 96%.^4^

A formação de pseudoaneurisma VE é uma CM ainda mais rara, com uma prevalência relatada de 0,05%.^5 , 6^ É uma consequência tardia de uma RPL não descoberta ou não operada do VE, formada quando a ruptura do miocárdio é contida por uma camada aderente de pericárdio, tecido cicatricial ou formação de coágulos.^2^ Como resultado, o evento inicial é tipicamente autolimitado e o sangramento causa um hemopericárdio não manifestado por tamponamento cardíaco.^2^ A cirurgia urgente é indicada um vez que pseudoaneurismas não tratados têm risco de ruptura de 30 a 45% e taxa de mortalidade de 50%.^3 , 5^

No caso clínico relatado, descreveu-se uma forma atípica de RPL incompleta ou subaguda VE, resultando na formação de tamponamento cardíaco e pseudoaneurisma.

No que diz respeito ao diagnóstico, o ETT é o pilar da avaliação inicial das CM após IM.^2^ O derrame pericárdico é o principal achado ecocardiográfico na RPL VE.^7^ No entanto, nos casos de pseudoaneurisma, o ETT é diagnóstico em apenas 26% dos pacientes e o método padrão-ouro para a sua identificação é a ventriculografia.^8^ A RMC é uma alternativa confiável quando o diagnóstico de pseudoaneurisma não pode ser estabelecido por nenhum dos métodos anteriores, conforme ilustrado no relato de caso apresentado. Ele identifica e distingue com precisão aneurismas falsos (pseudoaneurisma) e verdadeiros.^9 - 11^ Os pseudoaneurismas geralmente envolvem paredes laterais ou inferiores; não possuem elementos do miocárdio e são caracterizados por um colo estreito (razão entre o diâmetro máximo do orifício e o diâmetro interno máximo da cavidade de 0,25-0,5) e uma transição abrupta do miocárdio normal para o defeito.^9 - 11^ Aneurismas verdadeiros são mais comuns em localização apical, anterior ou anterolateral; contêm elementos do miocárdio e incluem pescoço largo (razão de diâmetros de 0,9-1,0) e uma transição suave do miocárdio normal para o miocárdio cicatricial.^9 - 11^ As características diferenciadoras anteriores presentes na RMC foram cruciais para o diagnóstico final da nossa paciente.

A pericardiocentese de emergência também permite o diagnóstico da RPL VE, quando um hemopericárdio está presente. Nesses casos, geralmente é observada coagulação espontânea do líquido pericárdico, devido ao consumo e exaustão dos fatores fibrinolíticos e anticoagulantes do mesotélio pericárdico.^12^ Sua ausência não exclui o diagnóstico, mas outras causas devem ser consideradas,^12^ conforme ilustrado pelo caso clínico relatado.

Para finalizar, nenhuma terapia de reperfusão imediata foi realizada nesse paciente, pois a ACI não mostrou lesão oclusiva aparente. A estabilização hemodinâmica foi prioridade. Posteriormente, apesar da falta de viabilidade do segmento envolvido demonstrado pela RMC, o ramo posterolateral foi revascularizado por ser considerado responsável pela perfusão de segmentos não necróticos.

## Conclusão

Este caso clínico demonstra uma CM extremamente rara após IM: uma forma atípica de RPL incompleta ou subaguda VE, resultando em formação de tamponamento cardíaco e pseudoaneurisma. Ele ilustra também como é difícil estabelecer o diagnóstico diferencial de dor no peito com instabilidade hemodinâmica e a etiologia de um tamponamento cardíaco. Por fim, destaca-se a versatilidade e a crescente aplicabilidade da RMC.
