# Supplementary material for: Uma Forma Complicada de Infarto de “Um Segmento” Miocárdico: O Papel da Imagem Cardiovascular
Source: Arq Bras Cardiol. 2020 Sep 11;115(1 Suppl 1):25–30. [Article in Portuguese] doi: 10.36660/abc.20190323 (PMC8386952; doi:10.36660/abc.20190323)
Supplement: Translation

# Translation: A Complicated “One Segment” Myocardial Infarction: The Role of Cardiovascular Imaging

**Authors:** Ana Rita Pereira, Ana Rita Almeida, Inês Cruz, Luis Rocha Lopes, Maria José Loureiro, Hélder Pereira

**Affiliations:** 1 Portugal Cardiology Department, Hospital Garcia de Orta EPE, Almada - Portugal; 2 England Barts Heart Centre, Barts Health NHS Trust, London - England; 3 England Institute of Cardiovascular Science, University College London, London - England; 4 Portugal Cardiovascular Center, Lisbon Academic Medical Center, University of Lisbon, Lisbon - Portugal

**Keywords:** Heart Rupture, Myocardial Infarction, Aneurysm,False, Diagnostic, Imaging, Echocardiography/methods

## Introduction

The incidence of mechanical complications (MC) after myocardial infarction (MI) was reduced to less than 1% with the routine use of primary reperfusion therapies.^1^MC are classified as early, including acute and subacute forms, and late or chronic.^2^The former mostly present as cardiogenic shock^2^and the latter may vary from asymptomatic to sudden death.^3^As all of these conditions may have potentially lethal consequences, timely diagnosis and treatment is necessary.^1 - 3^

## Case Report

A 57-year-old woman with smoking habits was admitted to the Emergency Department with oppressive anterior chest pain, nausea, and vomiting. Four days earlier, the patient reported similar symptoms with hours of evolution but spontaneous relief. Upon admission, she was conscious and maintained chest pain. Medical examination revealed hypotension, tachycardia, polypnea, and signs of decreased peripheral perfusion.

A 12-lead electrocardiogram showed sinus tachycardia with a 4 mm ST-segment elevation in DI and aVL leads, and a 4 mm ST-segment depression in inferior leads. Additional work-up revealed lactic acidosis, elevated systemic inflammatory parameters, and increased myocardial necrosis markers. Transthoracic echocardiogram (TTE) demonstrated a hypertrophic and non-dilated left ventricle with lateral hypokinesia, but with preserved systolic function; a moderate pericardial effusion with partial diastolic collapse of the right cavities; and a dilated inferior vena cava without respiratory variation ( Figure 1 ; Video 1). There were no significant valvular findings and the aortic root and arch were normal.

**Figure 1:** – Transthoracic echocardiogram. (A) Parasternal long-axis and (B) Parasternal short-axis views showing a mild hypertrophic and non-dilated left ventricle; (C) Apical 4-chamber view showing a partial collapse of right cavities and a lateral hypokinesia but preserved systolic function of the left ventricle; (D) Subcostal view showing a dilated inferior vena cava, with no respiratory size variation. The yellow arrow indicates a moderate pericardial effusion. LV: left ventricle; LA: left atrium; Ao: Ascending aorta; RV: right ventricle; RA: right atrium; IVC: inferior vena cava

**Video 1:** – Transthoracic echocardiogram, four-chamber view, showing a non-dilated left ventricle with lateral hypokinesia and a moderate pericardial effusion with partial diastolic collapse of right cavities. URL: http://abccardiol.org/supplementary-material/2020/11503/2019-0853_video01.mp4

Due to suspicion of subacute ST-elevation myocardial infarction (STEMI) complicated by left ventricular (LV) free wall rupture (FWR), no anti-thrombotic medication was administered, and the patient was submitted to an invasive coronary angiography (ICA) and ventriculography (Video 2). A 90% stenosis of the posterolateral branch was observed, although apparently no occlusive lesion, ventricular rupture or segmental wall motion abnormalities were found.

**Video 2:** – Ventriculography showing no apparent ventricular rupture or segmental wall motion abnormalities. URL: http://abccardiol.org/supplementary-material/2020/11503/2019-0853_video02.mp4

After angiography, her clinical status worsened. Cardiac tamponade was admitted and an emergency percutaneous pericardiocentesis was performed with drainage of 200 mL of hematic fluid with no spontaneous coagulation, resulting in global improvement ( Figure 2 ). Fluid analysis revealed an exudate and normal adenosine deaminase. Microbiological analyses were negative and the cytological test did not reveal neoplastic cells. In order to determine the effusion aetiology, viral serologies, autoimmunity, and thoracoabdominal-pelvic computed tomography were also performed with normal results.

**Figure 2:** – Drainage system of percutaneous pericardiocentesis with around 200 mL of hematic pericardial fluid.

Given the absence of a specific diagnosis, a cardiac magnetic resonance imaging (CMR) was performed eight days after hospital admission. It revealed dyskinesia of the mid-segment of the lateral wall in the cine sequences, transmural hyperintense sign in the T2-weighted short-tau inversion recovery images ( Figure 3A and 3B ) — compatible with oedema — and late transmural enhancement ( Figure 3C and 3D ) — suggesting myocardial necrosis — of this segment. These findings were compatible with subacute MI of the mid-segment of the lateral wall with no apparent viability. Moreover, the absence of myocardial tissue between the mid-segments of the lateral and inferolateral walls was observed, surrounded by a small saccular protuberance with a narrow neck, suggesting a pseudoaneurysm at that location ( Figure 3E and 3F ; Video 3).

**Video 3:** – Cardiac magnetic resonance imaging, steady-state free precession cine images, sequentially short-axis, four-chamber and three-chamber views, showing dyskinesia of the mid-segment of the lateral wall and a saccular protuberance between the mid-segments of the lateral and inferolateral walls, suggesting a pseudoaneurysm. URL: http://abccardiol.org/supplementary-material/2020/11503/2019-0853_video03.mp4

Thus, the initially suspected diagnosis was confirmed: subacute STEMI complicated with LV FWR that evolved to cardiac tamponade and posteriorly to a pseudoaneurysm formation. Due to the risk of fatal complications, the patient was submitted to cardiac surgery. With no need for cardiopulmonary bypass, a coronary artery bypass grafting with saphenous vein graph to the posterolateral artery and a pseudoaneurysm plication were performed. Currently, she is asymptomatic.

## Discussion

FWR is an uncommon and early MC of MI, with a reported incidence of less than 1%.^2^There are two clinical groups: the “blow-out type” (complete or acute rupture) with a macroscopic defect and high-volume bleeding, leading to cardiac tamponade; and the “oozing type” (incomplete or subacute rupture) without an obvious bleeding source and slow blood accumulation.^2^The latter type corresponds to up to one-third of the cases and can progress to complete rupture or pseudoaneurysm formation.^1 - 3^In both types, immediate surgery is vital, as FWR has a mortality rate ranging between 60 and 96%.^4^

LV pseudoaneurysm formation is an even rarer MC, with a reported prevalence of 0.05%.^5 , 6^It is a late consequence of an undiscovered or unoperated LV FWR, formed when the myocardial rupture is contained by an adherent layer of pericardium, scar tissue or clot formation.^2^As a result, the initial event is typically self-limited and bleeding causes an hemopericardium not manifested by cardiac tamponade.^2^Urgent surgery is indicated as untreated pseudoaneurysms have a 30 to 45% risk of rupture and a mortality rate of 50%.^3 , 5^

In the reported clinical case, an atypical form of incomplete or subacute LV FWR resulting in both cardiac tamponade and pseudoaneurysm formation was described.

Concerning diagnosis, TTE is the cornerstone for the initial evaluation of MC following MI.^2^Pericardial effusion is the main echocardiographic finding in LV FWR.^7^However, in cases of pseudoaneurysm, TTE is diagnostic in only 26% of patients and the gold standard method, for its identification is ventriculography.^8^When pseudoaneurysm diagnosis cannot be established by any of the previous methods, CMR is a reliable alternative, as illustrated by the presented case report. It accurately identifies pseudoaneurysms and distinguishes them from true aneurysms.^9 - 11^Pseudoaneurysms, or false aneurysms, commonly involve lateral or inferior walls; they have no myocardial elements and are characterized by a narrow neck (ratio of the maximum diameter of the orifice to the maximum internal diameter of the cavity of 0.25-0.5) and an abrupt transition from normal myocardium to the defect.^9 - 11^True aneurysms are more common in apical, anterior or anterolateral locations; contain elements of myocardium and include a wide neck (diameters ratio of 0.9-1.0) and a smooth transition from normal to thinned myocardium.^9 - 11^The previous differentiating features presented in the CMR were crucial for the final diagnosis of our patient.

Emergency pericardiocentesis also enables the diagnosis of the LV FWR, when an hemopericardium is present. In these cases, spontaneous coagulation of pericardial fluid is usually observed, due to the overwhelming of the fibrinolytic and anticlotting activities of the pericardial mesothelium.^12^Its absence does not exclude the diagnosis, but other causes must be considered,^12^as illustrated by the reported clinical case.

Finally, no immediate reperfusion therapy was performed on this patient, since ICA did not show any apparent occlusive lesion. Hemodynamic stabilization was the priority. Later, despite the lack of viability of the involved segment demonstrated by CMR, the posterolateral branch was revascularized as it was considered responsible for perfusion of non-necrotic segments.

## Conclusion

This clinical case demonstrates an extremely rare MC following MI: an atypical form of incomplete or subacute LV FWR resulting in both cardiac tamponade and pseudoaneurysm formation. It also illustrates how difficult it can be to establish the differential diagnosis of chest pain with hemodynamic instability, and the etiology of a cardiac tamponade. Ultimately, it highlights the versatility and increasing applicability of CMR.

**Figure 3:** – Cardiac magnetic resonance imaging. (A) Four-chamber and (B) short-axis views, T2-weighted short-tau inversion recovery (STIR) images, showing transmural hyperintense sign of the mid-segment of the lateral wall compatible with oedema; (C) Four-chamber and (D) short-axis views, late gadolinium enhancement images, showing contrast enhancement of the same segment, suggesting myocardial necrosis; (E) End-diastolic and (F) end-systolic phases, steady-state free precession (SSFP) cine images, showing dyskinesia of the mid-segment of lateral wall and a saccular protuberance suggesting a pseudoaneurysm (yellow arrow).
